# iTRAQ-based quantitative proteomic analysis reveals important metabolic pathways for arsenic-induced liver fibrosis in rats

**DOI:** 10.1038/s41598-018-21580-x

**Published:** 2018-02-19

**Authors:** Shunhua Wu, Jing Li, Xiang Jin

**Affiliations:** 1 Department of Occupational and Environmental health, School of public health, Xinjiang Medical University, Urumqi, 830011 China; 2 Shenzhen Omics Medical Research Center, Shenzhen, 518053 China

## Abstract

Long-term consumption of sodium arsenite contaminated water can cause endemic arsenic disease. The proteome profile changes of liver fibrosis after exposure to arsenite containing water remain unclear. In this study, Sprague-Dawley (SD) male rats were treated with sodium arsenite (iAs3+), using a daily dose of 1.36 mg/kg body weight (medium dose group, M), 2.73 mg/kg body weight (high dose group, H) or deionized water (control group, C). Isobaric tags for relative and absolute quantitation (iTRAQ) were used to identify the different abundant proteins (DAPs) after arsenic-induced liver fibrosis. A total of 2987 high-quality proteins were detected (95% confident peptides ≥ 2), 608 of which were differentially expressed (fold change > 2 and *p* < 0.05) in M group and 475 in H group. Moreover, 431 DAPs were found in both M and H groups and used in subsequent bioinformatic analyses. Gene ontology (GO) analysis revealed 4,709 GO terms could be mapped, among which purine binding, actin filament binding and protein kinase binding were the most enriched terms for molecular function category. In addition, protein-protein interaction analysis showed six clusters of interaction networks. Our data provided new insights into the proteome changes after arsenic-induced liver fibrosis in model rats.

## Introduction

In nature, trivalent arsenite [As (III)] is the most common oxidation states for soluble arsenic^1^. As (III) toxicity is due to its ability to covalently bind protein sulfhydryl groups present in proteins, which therefore results in inhibition of many enzymes that hold the critical positions^2^. International Agency for Research on Cancer had determined that Arsenic is a toxic metalloid and classified as a group 1 carcinogen^3^. During the last decade, increasing evidence indicates that arsenic exposure is related with cardiovascular disease^4^, diabetes mellitus^5^, neurological disorders^6^ and reproductive effects^7^. Furthermore, arsenic has been linked with the onset and progression of tumors in many organs, including lung, liver, kidney, skin and bladder^8,9^. Arsenic exposure can occur via various routes and among those, contaminated drinking water is one of the major threats to human health^10^. It has been described that about 19.6 million people are at risk of arsenic-contaminated ground water in China^11^. In microorganisms, mechanism of As (III) has been clearly studied. As (III) are the analogues of glycerol and phosphate and enters microbial cells via glycerol transporters (GlpF)^1^. Intracellular As (III) serves as an inducer to bind the regulatory protein ArsR that departs from the operator and transcribe genes. The intracellular As (III) is pumped out via three different ways: 1) by ArsB, 2) byACR3 or 3) by the ArsA/ArsB complex with the interaction of ArsD. In addition to direct extrusion, the intracellular As (III) can be methylated by ArsM and pumped out of the cell by an unidentified transporter^12^. However, the molecular mechanisms of arsenic toxicity remain obscure in mammals.

Proteomics is the large-scale study of proteins and employs techniques to identify complete protein complements of the expressed genome, which provides a macroscopic view of what is expressed and present under different growth conditions, thereby enabling more constructive targeted experimentations. Proteomics in comparison to genomics is an ideal tool to discover different abundant proteins, as they reflect the actual activity with respect to metabolic reactions and regulatory cascades and provide more direct information about microbial activity than functional genes and even their corresponding messenger RNAs^13^. Isobaric tags for relative and absolute quantification (iTRAQ) are employed widely, with a proven value in discovery-based proteomics^14,15^. This technology allows for simultaneous protein identification and (relative) quantification obtained at the MS/MS level from peptide fragments and low mass reporter ions. Meanwhile, iTRAQ is conceptually elegant, since peptides are labelled at the N-terminus and at the ε-side chain of lysines^16–18^. Moreover, iTRAQ is robust, due to the stable N-hydroxysuccinimide (NHS) ester chemistry, and has been implemented widely^15^. The ability to simultaneously analysis multiple samples has proven popular, and iTRAQ now has applicability across diverse MS platforms, including Q-TOF, ion trap and others^19,20^. Currently, iTRAQ has especial advantages over conventional proteomics techniques because this one identifies and quantifies many proteins from the specific biological environments using label peptides able to be identified by sensitive mass spectrometers. The iTRAQ analysis can be further enhanced with use of robust bioinformatic tools^21^ and statistical analysis^22^, which support the biological evidence discovered.

In this work, we performed an iTRAQ-based quantitative proteomic analysis on Sprague-Dawley (SD) male rats with specific pathogen free (SPF) under different exogenous sodium arsenite (iAs^3+^) treatments. Among 2987 identified proteins, 608 of were considered as differentially expressed in M group and 475 in H group. Moreover, 431 common DAPs were found in both M and H groups and used in subsequent bioinformatic analyses. A total of 4,709 GO terms could be mapped, among which purine binding, actin filament binding and protein kinase binding were the most enriched terms for molecular function category. The protein-protein interaction analysis indicated six clusters of interaction networks. Our results provide new insights into the proteome profile changes of liver fibrosis induced by sodium arsenite stress.

## Method and Materials

### Ethics statement

This study was approved by the Institutional Animal Ethics Committee of Institute of first affiliated hospital of Xinjiang Medical hospital (No. 20120220-140) and all methods were performed in accordance with the relevant guidelines.

### Animals and arsenic treatment

This study was approved by the Institutional Animal Ethics Committee of Institute of first affiliated hospital of Xinjiang Medical hospital (No. 20120220-140). All animals were carefully handled in compliance with guidelines. Thirty male Sprague–Dawley rats weighing approximately 100 ± 20 g were supplied by laboratory animal center of Xinjiang University. The acute oral toxicity test in rat was performed and LD50 was determined using Horn’s method (41 mg/kg). The administration dose of 1/15 (41 mg/kg) (2.73 mg/kg) was considered as high dose, and 1/30 of (41 mg/kg) (1.36 mg/kg) was considered as medium dose^23^. These rats were randomly divided into three groups after one week of adaptation breeding: ten for control group, ten for medium dose groups, and ten for high dose. The animals were exposed to arsenic via drinking water; the controls received deionized water. Animals had access to both water and food ad libitum throughout. After the exposure of six months, rats were sacrificed by laparotomy following anesthesia. Livers were carefully removed. The same regions of livers (about 1 g of each sample) were fixed by 4% paraformaldehyde solution for observation of light microscope and the same regions of livers (about 1 g of each sample) were fixed by 4% glutaraldehyde solution for observation of Transmission electron microscope (TEM). Haematoxylin Eosin (H&E) staining were prepared according to the previous method^24^. Samples were fixed in 10% buffered formalin, and embedded in paraffin. Three to five micrometer thick sections were stained with hematoxylin (Sigma, H3136) for 10 min and with eosin (Sigma, E4382) for 1 min to establish the diagnosis areas. Meanwhile, a double staining of uranyl acetate and lead citrate method was used according to the previous study^25^. The rest of all samples were immediately frozen by immersing into liquid nitrogen, and then stored at −80 °C until further processing.

### Protein extraction, digestion, and iTRAQ labeling

According to the liver physiological performance and analysis, we chose the groups of control, medium dose and high dose for further iTRAQ studies. Proteins from three biological replicates in each treatment were extracted using the phenol extraction method^26^. Each sample was extracted three times. About 200 μg of pooled protein samples were incorporated into 30 μL STD buffer (4% SDS, 100 mM DTT, 150 mM Tris-HCl, pH 8.0), incubated in boiling water for 5 min, cooled to room temperature, diluted with 200 μL UA buffer (8 M urea, 150 mM Tris-HCl, pH 8.0), and subjected to 30 kDa ultrafiltration. The samples were centrifuged at 14,000 × g for 15 min and 200 μL UA buffer was added and centrifuged for another 15 min. After adding 100 μL UA buffer (0.05 M iodoacetamide), the samples were incubated for 20 min in darkness, and then centrifuged for 10 min. After washing the filters three times with 100 μL UA buffer, 100 μL DS buffer (50 mM triethylammoniumbicarbonate at pH 8.5) was added to the filters and centrifuged for 10 min. This step was repeated twice and then 2 μg trypsin (Promega) in 40 μL DS buffer was added to each filter. The samples were incubated overnight at 37 °C. The resulting peptides were collected by centrifugation. The filters were rinsed with 40μL10 × DS buffer and centrifuged again. Finally, the peptide content was tested by spectral density with UV light at 280 nm. About 100 μg peptides of each treatment group were labeled with iTRAQ reagents following the manufacturer’s instructions (Applied Biosystems).

### Separation of peptides and LC mass spectrometric analysis

Prior to mass spectrometric analysis, peptides were purified from excess labeling reagent by High Performance Liquid Chromatography (HPLC) (Shimadzu Corporation). The labeled samples were dried and then diluted with 20-fold cation exchange binding buffer A (10 mM KH_2_PO_4_, pH 3.0, 25% ACN). The labeled samples were separated into ten fractions by a poly-sulfoethyl A column. A poly-sulfoethyl 4.6 × 100 mm A column (5 m, 200 A; Poly LC, Columbia, MD, USA) gradient elution was applied to separate peptides at a flow rate of 1 mL/min with elution buffer B (10 mM KH_2_PO_4_, pH 3.0, 500 mM KCl, 25% ACN). Eluted peptides were collected and desalted by an offline fraction collector and C18Cartridge (Sigma) prior to MS analysis. The MS analysis was performed using AB SCIEX Triple TOF 5600 mass spectrometer. Protein identification with two technical replicates for each biological replicate was performed. For protein identification, the peptide molecules were identified by ProteinPilot 4.5 (AB SCIEX) software, and the parameter sets were as follows: peptide mass tolerance 3 Da, fragment ion tolerance 1 Da, and number of allowed missed tryptic cleavage sites 2. The acquired data was automatically searched against the UniProt Database and quantitative analysis. The “Sample” labeled with control was used as reference. The final ratios of protein were then normalized by the medium average protein ratio for unequal mixes of different labeled samples. Thus, if samples from each experimental condition are not combined in exactly equal amounts, this normalization corrects the systematic error. Confidence threshold of protein (Unused ProtScore) was set as over 1.3. Meanwhile, at least 1 matched peptides within the 95% confidence interval for protein identification.

### Bioinformatic analyses

The heat map was generated using heatmap V2.0 (R-package). Gene ontology enrichment analysis of different abundant proteins was implemented by WEGO software (http://wego.genomics.org.cn/cgi-bin/wego/index.pl), in which gene length bias was corrected. False discovery corrected *p* value < 0.05 was chosen to include as much as possible items^27^. The KEGG ontology based annotation system (KOBAS) software were used to perform KEGG annotation and enrichment analysis (http://kobas.cbi.pku.edu.cn/home.do)^28^. The KEGG is a database resource for understanding high-level functions and utilities of the biological system, such as the cell, the organism and the ecosystem, from molecular-level information, especially large-scale molecular datasets generated by genome sequencing and other high-through put experimental technologies (http://www.genome.jp/kegg/)^29^. Gene ontology enrichment analysis software toolkit (GOEAST)^30^ and STRING (https://string-db.org/) were further used to analyze the GO term tree-view enrichment and protein-protein interaction networks, respectively.

## Results

### Histopathologic features

**Figure 1:**
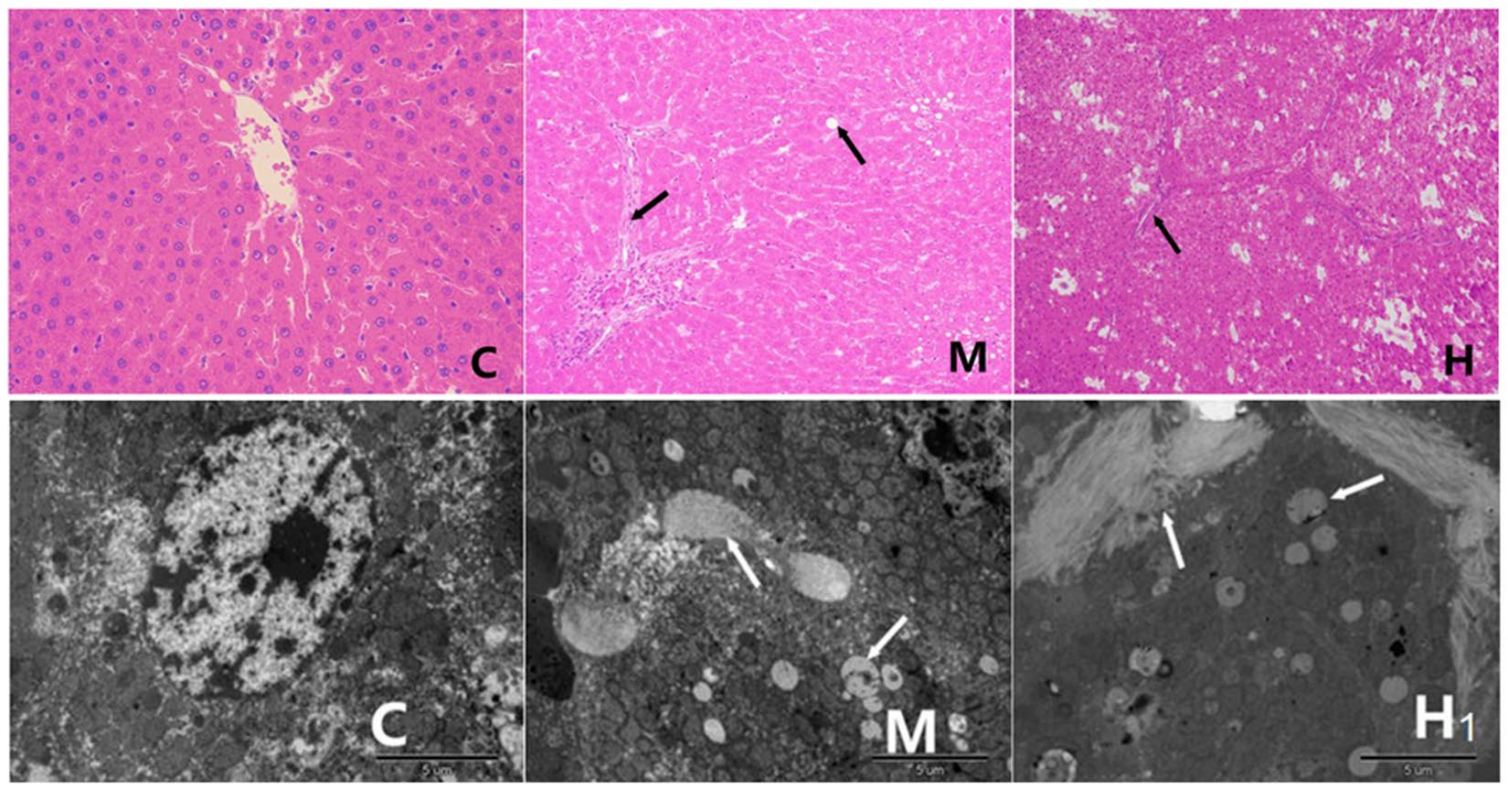
Representative appearances of histopathological (H&E) staining and transmission electron microscopy (TEM) of the liver with control, medium dose and high dose of sodium arsenite. In the subgroups of histopathological (H&E) staining, C means control group (deionized water feeding) with 200× magnification, M means group with medium dose of sodium arsenite with 200× magnification, H means group with high dose of sodium arsenite with 100× magnification. Similar way of grouping can be found in the subgroups of transmission electron microscopy (TEM), and scale bar is 5 µm.

The animals were exposed to arsenic via drinking water for 24 weeks. Figure 1 showed the representative appearances of histopathological (H&E) staining and transmission electron microscopy (TEM) of the liver with control, medium dose and high dose of sodium arsenite. In the subgroups of Haematoxylin Eosin (H&E) staining, the results of control group (deionized water feeding) indicated that the structural integrity of liver cells and none of edema, degeneration and necrosis observed in the cells surrounding central veins. The results of medium dose group of sodium arsenite suggested that there was an obviously effect on hemangiectasis and edema of liver cells. Meanwhile, hepatic steatosis can be clearly observed. Moreover, the results of high dose group of sodium arsenite showed that hemangiectasis was more obvious than that in medium dose group. Cell swelling, inflammatory cell infiltration and fiber hyperplasia and other unique cell trauma characters were apparently. In order to confirm the results mentioned above, we performed the observations of transmission electron microscopy (TEM). The results of TEM were consistent with the phenomenon of light microscope, degeneration and liver fibrosis had become into an inevitable trend as increasing the concentration of sodium arsenite breeding.

### Protein expression profiles under sodium arsenite stress

**Figure 2:**
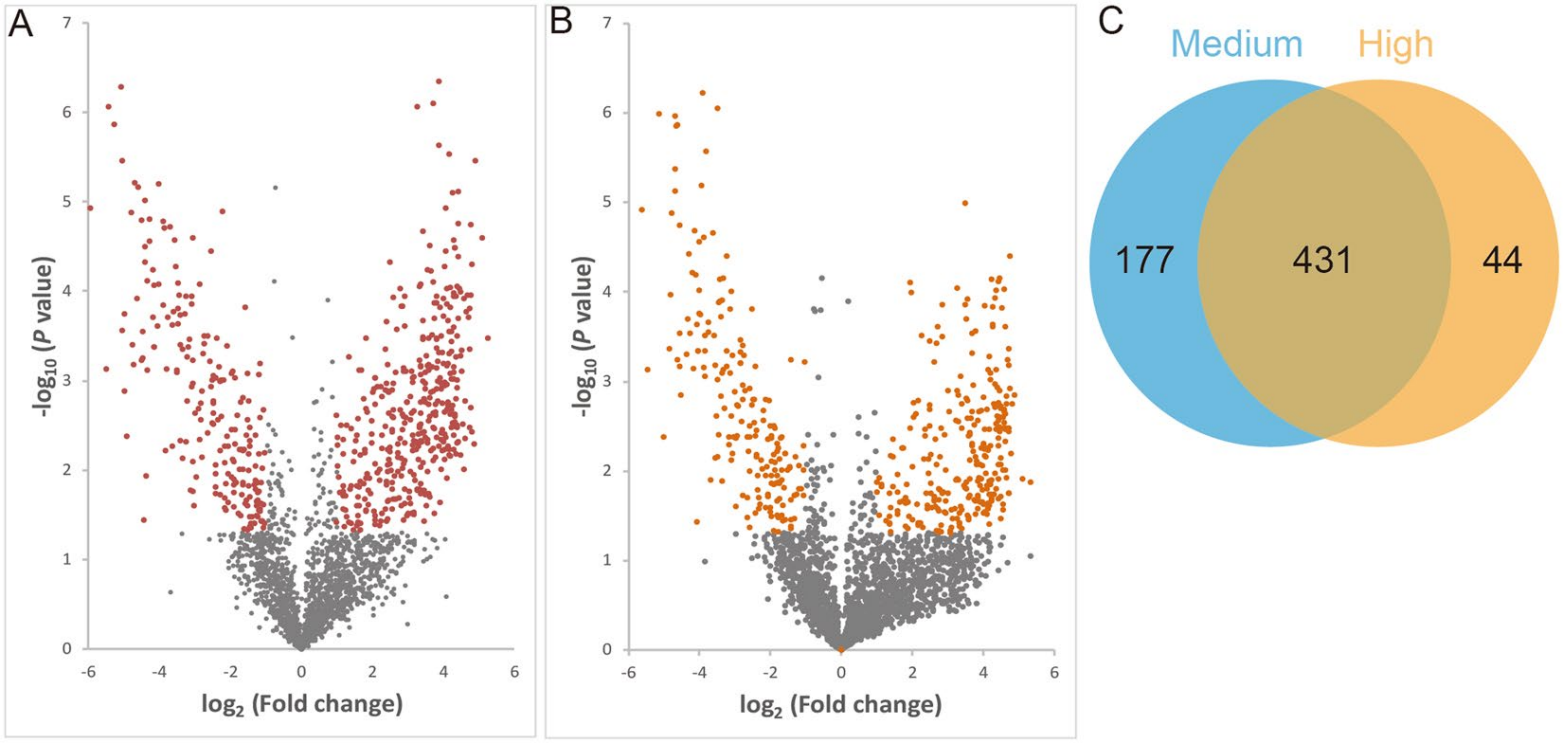
Venn diagrams of differentially expressed proteins that are detected in high dose group and medium dose group in this study. The overlap represents 431 differentially expressed proteins that are common in high dose group and medium dose group than in control group.

**Figure 3:**
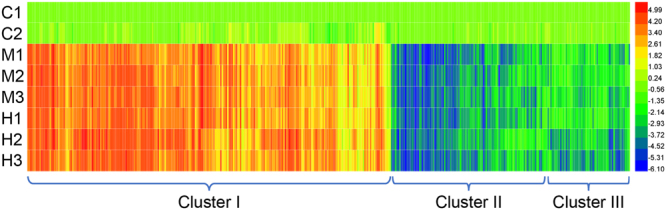
Hierarchical clustering of 431 identified differentially expressed proteins in three different treatments. Three main protein expression patterns were identified: Cluster I, the highest protein expression level occurring in medium or high dose treatment; Cluster II, the highest protein expression level occurring in the control group; Cluster III, the lowest protein expression level occurring in the high dose group. The color bar represents the log_2_ fold change of protein level is shown in the right.

The identified proteins were separated into homology groups by aligning all proteins and recursively grouping proteins with highly significant alignment scores. A total of 2987 high-quality proteins were detected (95% confident peptides ≥ 2). Further, 608 non-redundant proteins showed differentially expressed (fold change > 2 and *p* < 0.05) in medium dose group than in control group. Meanwhile, a total of 475 non-redundant proteins showed differentially expressed in high dose group than in control group. Moreover, 431 different abundant proteins that are common in high dose group and medium dose group (Fig. 2 and Table S1). Hierarchical cluster analysis according to Pearson’s distance indicated three groups with clearly different expression patterns among the 431 different abundant proteins (Fig. 3, Clusters I–III).

### Gene Ontology and KEGG pathway analysis of different abundant proteins

**Figure 4:**
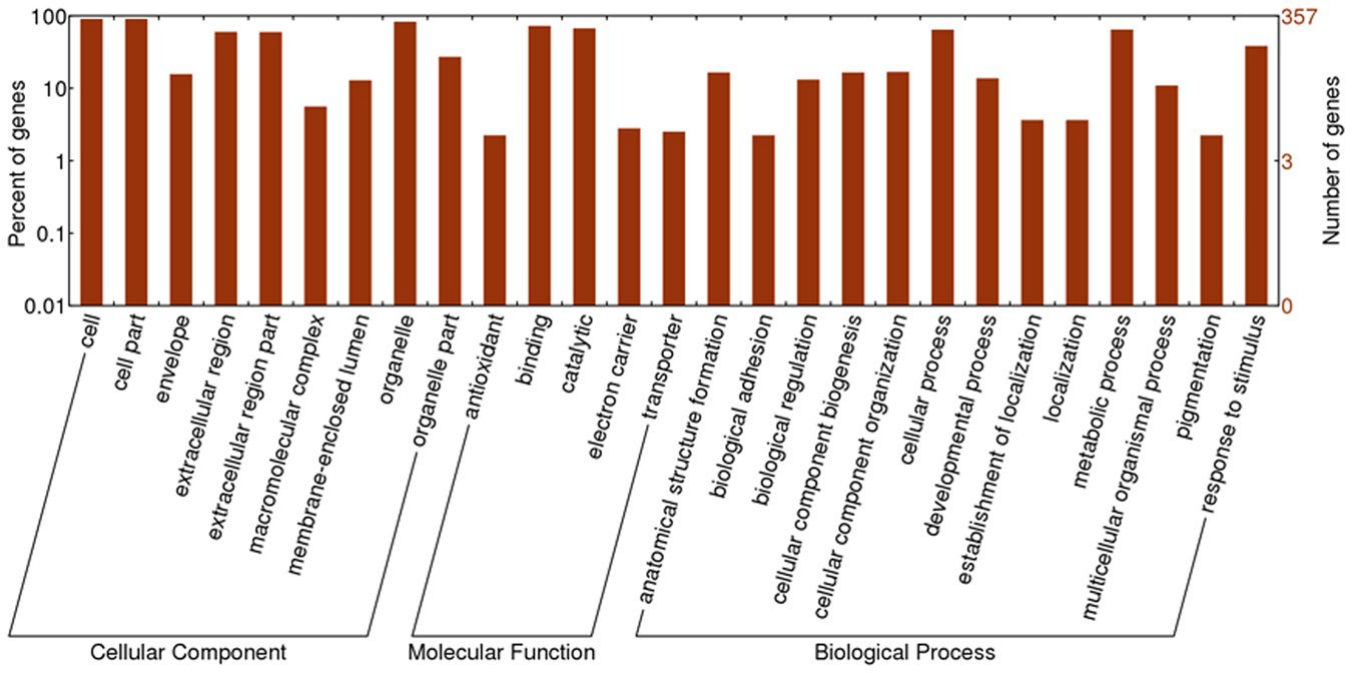
Gene ontology analysis of 431 differentially expressed proteins identified in this study.

Gene Ontology (GO) terms were further assigned to different abundant proteins based on their sequence similarities to known proteins in the UniProt database annotated with GO terms as well as InterPro and Pfam domains they contain. GO annotation and enrichment analysis of all 431 different abundant proteins was implemented by the KOBAS software and WEGO software, respectively, in which gene length bias was corrected. In this study, 431 differential expression genes involve 206 pathways (Table S2). Figure 4 shows the results of pathways enrichment, it clearly displays that metabolic pathways were the top enriched term. 157 differential expression genes that identified in our study participate in the metabolic pathways. Moreover, it is worth noting that Carbon metabolism, Biosynthesis of amino acids, Valine, leucine and isoleucine degradation and Fatty acid degradation were also significant enriched in this study. The pathways mentioned above were adopted with the function that liver played.

**Figure 5:**
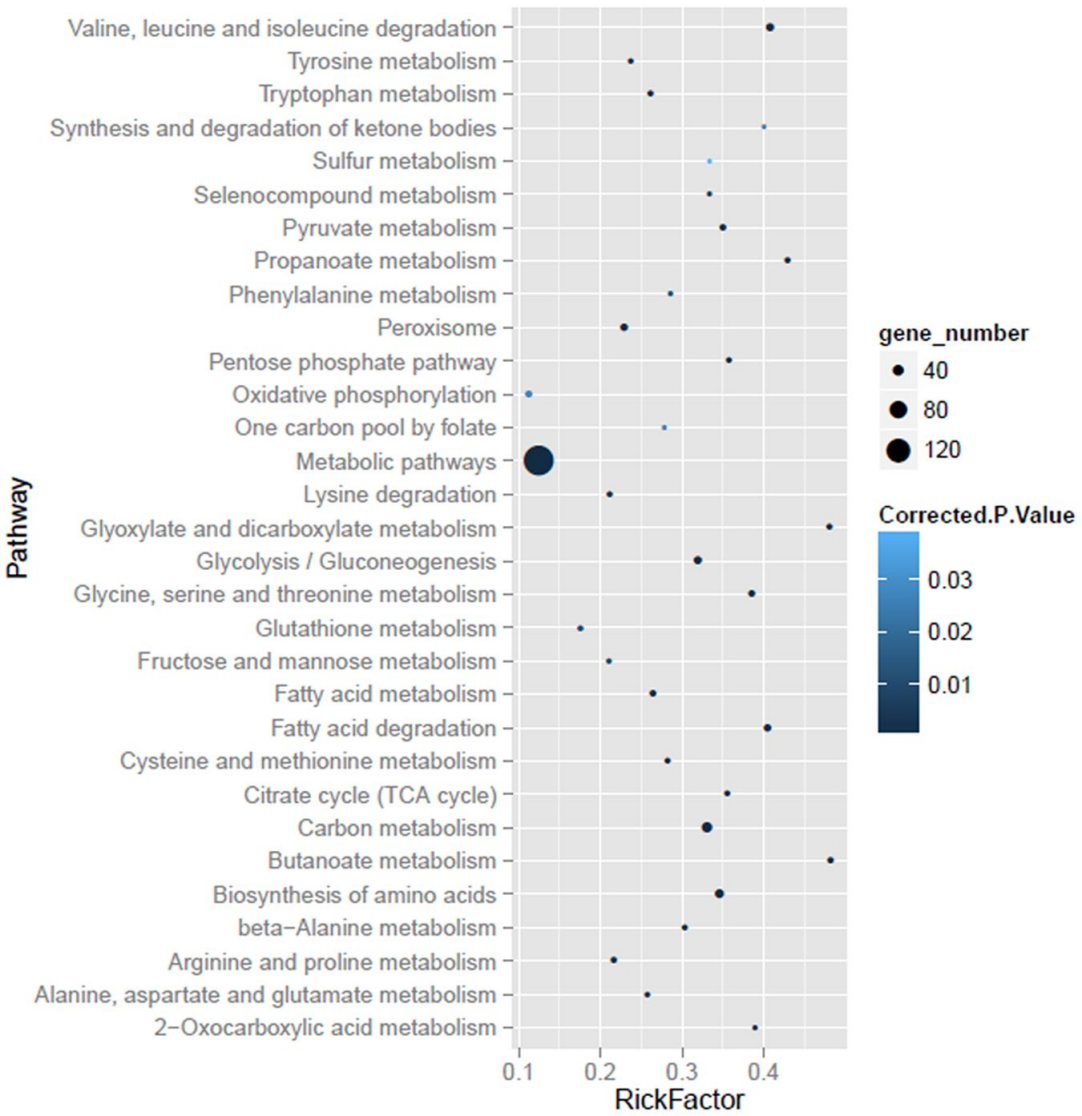
Scatter diagram of enriched KEGG pathways. Degree of enrichment was measured by Rich factor, Q value and the number of genes that enriched in one pathway. Rich factor means the ration of the number of differential expression genes that enriched in one pathway and the number of GO annotation. The greater value of Rich factor represents the higher degree of enrichment. Q value is a variant of P value, lower numbers equate to significant enrichment. Y-axis represents the name of pathway, X-axis represents the Rich factor. Point size means the number of differential expression genes in one pathway, and the color of point means the range of Q value.

GO terms with corrected *p* value less than 0.05 were considered significantly enriched by different abundant proteins. There were 4,709 GO items were harvested in this study. Organic acid metabolic process (GO: 0006082) and carboxylic acid metabolic process (GO: 0019752) were the two most enriched items. The distribution bar charts of the biological processes, cellular components, and molecular functions are shown in Fig. 5. From the perspective of biological processes, cellular process (GO: 0070062, Corrected *p*-Value: 1.65E-31), metabolic process (GO: 0006082, Corrected *p*-Value: 4.01E-37) and response to stimulus (GO: 0010038, Corrected *p*-Value: 9.30E-10) were the top three significantly enriched terms. From the cellular component perspective, cell (GO: 0042995, Corrected *p*-Value: 3.95E-05), cell part (GO: 0044464, Corrected *p*-Value: 0.99) and organelle (GO: 0065010, Corrected *p*-Value: 2.81E-34) were the top three significantly enriched terms. From the molecular function perspective, binding (GO: 0048037, Corrected *p*-Value: 3.40E-35) and catalytic (GO: 0003824, Corrected *p*-Value: 7.70E-13) were the top two significantly over-represented terms.

Pathways enrichment analysis can give some clues to the biochemical and signal transduction pathways that differential expression genes may participate in. KEGG is a database resource for understanding high-level functions and utilities of the biological system, such as the cell, the organism and the ecosystem, from molecular-level information, especially large-scale molecular datasets generated by genome sequencing and other high-through put experimental technologies (http://www.genome.jp/kegg/). We used KOBAS software to test the statistical enrichment of differential expression genes in KEGG pathways (Fig. 5).

### Pathway enrichment and protein-protein network analyses of DAPs

**Figure 6:**
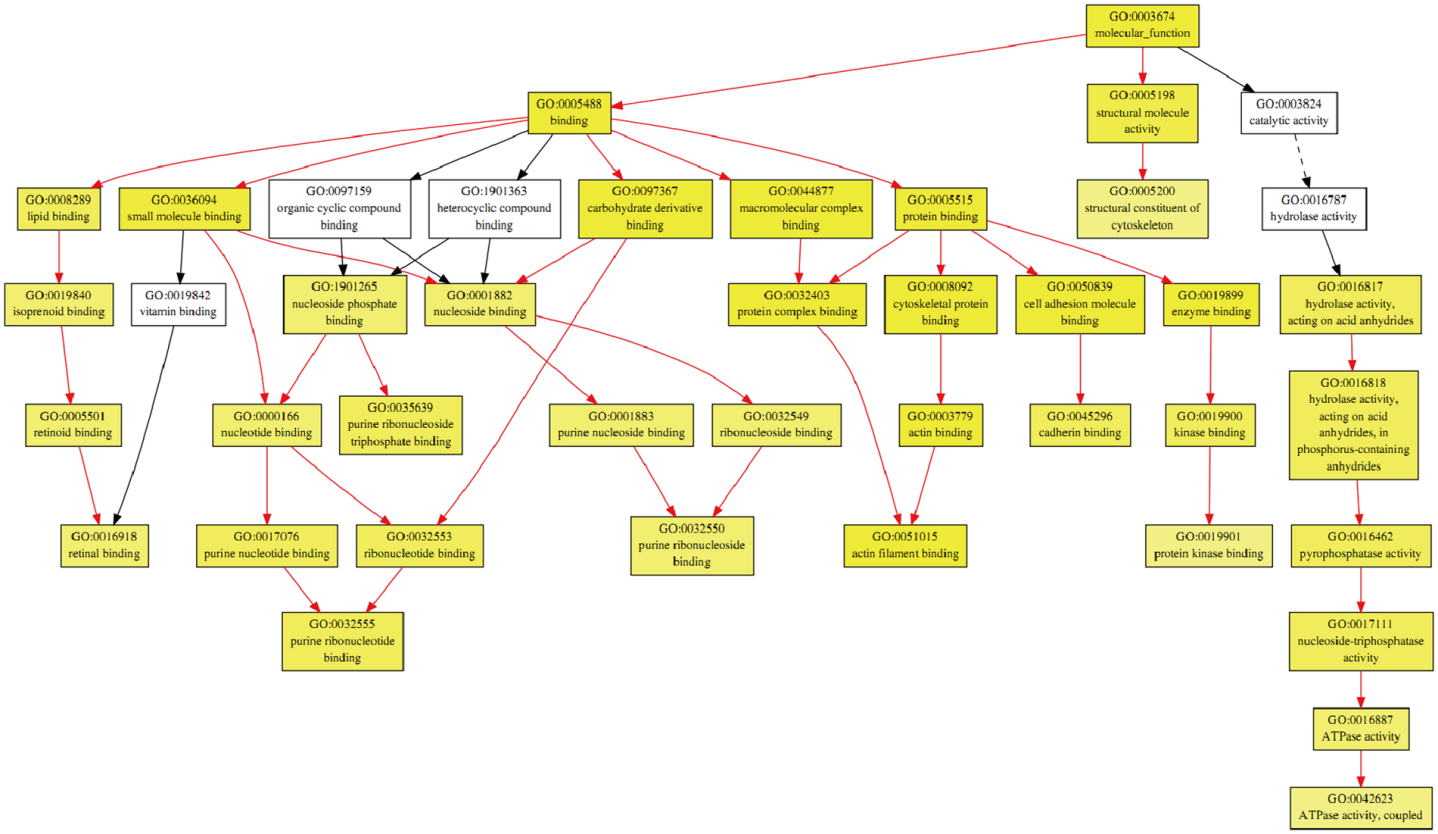
Tree-view of the GO enrichment analysis of molecular function category for the 171 proteins that decreased in both medium and high dose treatment. The GO terms which were significantly enriched (FDR *p* value < 0.001) were shown in colored boxes. The mature of yellow color correlate with the significance levels of each term. The meanings of arrow types are indicated: red arrows linked two significant terms; black arrows linked one significant term and one non-significant term; black dashed line arrows linked tow terms that non-significant.

Gene ontology pathway enrichment analysis was performed using gene ontology enrichment analysis software toolkit (GOEAST). The most significantly enriched branch-end term of the molecular function category for the 171 decreased proteins were actin filament binding, protein kinase binding and purine ribonucleotide binding (Fig. 6). Moreover, the tree-views of the biological process and cellular component categories for the 260 increased proteins and 171 decreased proteins were also provided as supplementary Figures S1–S5 because of the big file size.

**Figure 7:**
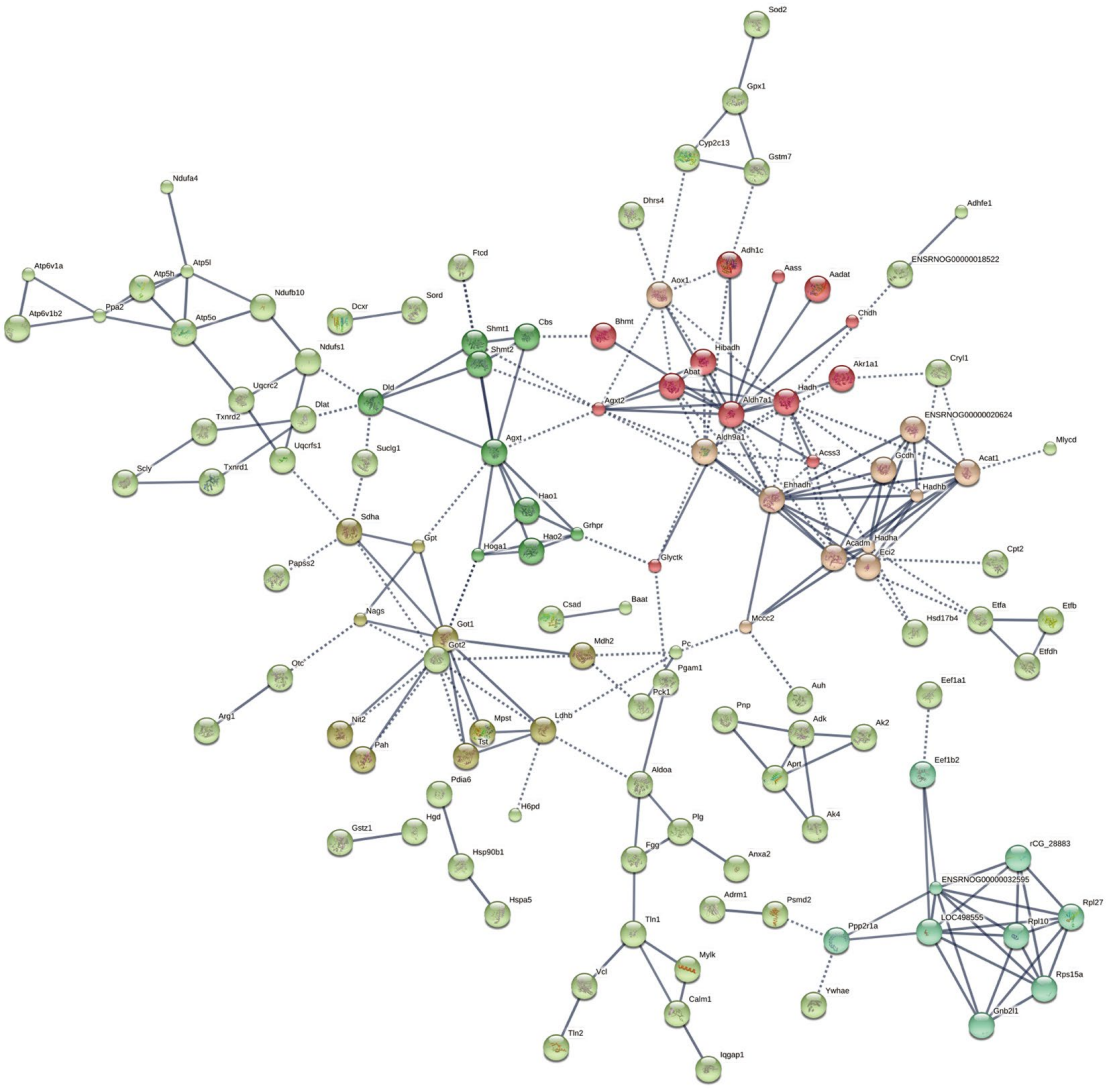
Network analysis of 431 CoDEPs. The protein-protein networks of the 431 CoDEPs were analyzed by STRING. The minimum required interaction score was set as the highest confidence (0.900) and the disconnected nodes in the network were hidden. The small nodes represent protein of unknown 3D structure and the large ones indicate the proteins with known or predicted 3D structure. The network map was clustered in 6 clusters using K-means method.

To determine the key proteins in the function network for the 431 common DAPs, online tool of STRING was used to analyze the protein-protein interaction networks. The results showed that 6 clusters were determined by K-means method, representing by different colored nodes (Fig. 7).

## Discussion

Several investigations have described how arsenic can alter gene expression or protein function by inducing oxidative stress and increasing mRNA expression in response pathways^31,32^. EGF receptor–mediated pathways can be activated by arsenic, leading to altered gene expression in mouse lung^33^. Zebra fish embryos exposed to 2 or 10 ppb arsenic showed decreased expression of genes involved in the innate immune response^34^. In addition, DNA methylation and histone acetylation can be altered by arsenic exposure^35,36^. These studies describe several mechanisms by which arsenic can influence gene expression.

Liver fibrosis is the excessive accumulation of extracellular matrix proteins including collagen that occurs in most types of chronic liver diseases. Advanced liver fibrosis results in cirrhosis, liver failure, and portal hypertension and often requires liver transplantation. Numerous works had been published on proteomics of liver fibrosis^37^. Several works reported iTRAQ-based proteomic research on liver fibrosis in rats induced by other factors, such as carbon tetrachloride^38^, dimethylnitrosamine^39^. However, the application of iTRAQ technologies to investigate the proteome changes after liver fibrosis induced by exposure to arsenite has not been reported before. Our work for the first time provides iTRAQ-based high-throughput proteomics data of arsenic-induced liver fibrosis in rats.

In this study, we performed an iTRAQ-based quantitative proteomic analysis on Sprague-Dawley (SD) male rats with Specefic pathogen Free (SPF) under different exogenous sodium arsenite (iAs^3+^) treatments. Differential expression analysis of control treatment and high dose and medium dose treatment was performed. We had noticed two interested places. Firstly, the transforming growth factor (TGF)-β1 plays a crucial role in the induction of the epithelial-to-mesenchymal transition (EMT) in hepatocytes, which contributes to the pathogenesis of liver fibrosis. The inhibition of the TGF-β1 cascade suppresses EMT and the resultant fibrosis^40^. The up-regulated expression of Hic-5 protein, which is encoded by TGF-β1 gene, in high dose treatment than in control treatment indicates the activation of TGF-β1 cascade signaling. TGF-β1 can significant promoted the accumulation of stimulating extracellular matrix (ECM) production, which may be the important reason that liver fibrosis. Secondly, as an antioxidant, glutathione (GSH) is essential for activation of redox system. GSH can active thiol enzyme so that it can achieve the aim of detoxication and maintain the biological function of cells. Moreover, GSH play an important role in liver-protection through transmethylation and transimidation reactions. GSH can build relationship with the protein products of AS3MT gene in the arsenic methylation metabolism. Inorganic arsenic can turn into exogenous dimethylarsinic acid (DMA) through methylated modification which needs consuming GSH and SAM, so that the content of GSH was decreased in blood and liver^41^.

Moreover, we identified 18 kinds of proteins that were up-regulated in high dose treatment and medium dose treatment than in control treatment which were related with glutathione metabolism. Of the 18 kinds of proteins, Gsta1, Gsta4, Gsta5, Gstt1, Gstt2, Gstk1, Gstp1, Gstm1, Gstm2, Gstm3, Gss, Gpx1, Gpx4, Esd, Hagh, Glo1, Mgst1 and B6DYQ5 were related with catalytic reaction of GSH and R-X, which can result in the transportation of arsenic extracellular. Gss, Esd, Hagh, Glo1 and Mgst1 were related with high expression of the enzymes that can promote the synthesis of glutathione (Table S1). Up-regulated expression of enzymes that promote the synthesis of glutathione may reflect the heavy demand of GSH. We speculated that the biosynthesis of GSH was enhanced under sodium arsenite stress. Therefore, the content of GSH should be excessive consumed. The results mentioned above suggested there were significant differences in liver fibrosis metabolism between sodium arsenite stress and control treatment in rat.

The differential abundant proteins were annotated by GO analysis, and thus explore the function of different abundant proteins. As expected, several GO terms including organic acid metabolic process, carboxylic acid metabolic process, oxoacid metabolic process, cofactor binding, extracellular vesicular exosome, extracellular vesicle and extracellular membrane-bounded organelle were significantly enriched. Most of the different abundant proteins were associated with metabolic processes. Furthermore, KEGG analysis showed 431 differential expression genes involved 206 pathways. It clearly displays that metabolic pathways were the top enriched term. 157 differential expression proteins that identified in our study participate in the metabolic pathways. Moreover, it is worth noting that carbon metabolism, biosynthesis of amino acids, valine, leucine and isoleucine degradation and fatty acid degradation were also significant enriched in this study. One of the significantly enriched pathways of differential expression proteins in the lipid accumulation was PPAR signaling pathway. Recent studies proved that PPARs functions as a key regulator of adipocyte differentiation, accumulation and phenotypes^42^. Genes enriched in the pathway were involved in lipid metabolisms including F Ilk, Ehhadh, Acadm, Hmgcs2, Dbi, Acsl1, Cpt2, Pck1 and Acox1. Most of them were involved in the stimulating extracellular matrix (ECM) production. PPARs (peroxisome proliferators-activated receptors) are known to play a key role in the metabolic pathways involving fatty acid oxidation and lipid metabolism^43^.

Therefore, our results supported that stimulating extracellular matrix (ECM) production were more enriched in medium dose and high dose treatments that in control group. Another enriched term of differential expression proteins was drug metabolism-cytochrome P450 signaling pathway. Cyp2c13, Adh1, Gstm1, Gstm2, Gstp1, Gsta5, Aox1, Gstm3 and Gstz1 were included in this pathway, which was adapted to the glutathione metabolism mentioned above (Table S3). The pathways mentioned above were adopted with the functions that hepatic fibrosis. In conclusion, sodium arsenite stress resulted in significant changes in proteins expression profiles in liver. Our results found that lots of proteins could be involved in the hepatic fibrosis resulting from sodium arsenite stress in male rats. Meanwhile, it provides a new clue for understanding the changes of the hepatic fibrosis mechanisms in rats under sodium arsenite stress.

## Conclusion

In summary, we demonstrated that profile of proteins expressed under sodium arsenic stress. A total of 2,987 proteins were detected, 608 of which were differentially expressed in M group and 475 in H group. Moreover, 431 DAPs were found in both M and H groups and used in subsequent bioinformatic analyses. Bioinformatic analyses revealed the enriched gene ontology and KEGG ontology terms. In addition, protein-protein interaction analysis showed six clusters of interaction networks. Notably, AS3MT, GSH and TGF-β1 which were considered associated with liver fibrosis were significant up-regulated under high dose sodium arsenic exposure. This work presented here would provide the detailed understanding on the mechanisms of liver fibrosis in rat under sodium arsenic stress.

## Electronic supplementary material

FigureS1-S5

TableS1-S3
