# Supplementary material for: iTRAQ-based quantitative proteomic analysis reveals important metabolic pathways for arsenic-induced liver fibrosis in rats
Source: Sci Rep. 2018 Feb 19;8:3267. doi: 10.1038/s41598-018-21580-x (PMC5818499; doi:10.1038/s41598-018-21580-x)
Supplement: Supplementary file 1 — FigureS1-S5 [file 41598_2018_21580_MOESM1_ESM.pdf]

iTRAQ-based quantitative proteomic analysis reveals important metabolic pathways for arsenic-induced liver fibrosis in rats

Shunhua Wu[1]*, Jing Li[1], Xiang Jin[1,2]

1. Department of Occupational and Environmental health, School of public health, Xinjiang Medical University, Urumqi, 830011, China

2. Shenzhen Omics Medical Research Center, Shenzhen, 518053, China

Running title: proteomic analysis of rat liver under arsenite stress

Supplementary Information

Supplementary Table S1. 431 differentially expressed proteins that is common in high dose group and medium dose group than in control group.

Supplementary Table S2. KEGG analysis of 431 differentially expressed genes

Supplementary Table S3. Gene ontology annotation of the 431 differentially expressed genes.

Supplementary Figure S1. Tree-view of the GO enrichment analysis of biological process category for the 171 proteins that decreased in both medium and high dose treatment.

Supplementary Figure S2. Tree-view of the GO enrichment analysis of biological process category for the 260 proteins that increased in both medium and high dose treatment.

Supplementary Figure S3. Tree-view of the GO enrichment analysis of cellular component category for the 171 proteins that decreased in both medium and high dose treatment.

Supplementary Figure S4. Tree-view of the GO enrichment analysis of cellular component category for the 260 proteins that increased in both medium and high dose treatment.

Supplementary Figure S5. Tree-view of the GO enrichment analysis of molecular function category for the 260 proteins that increased in both medium and high dose treatment.

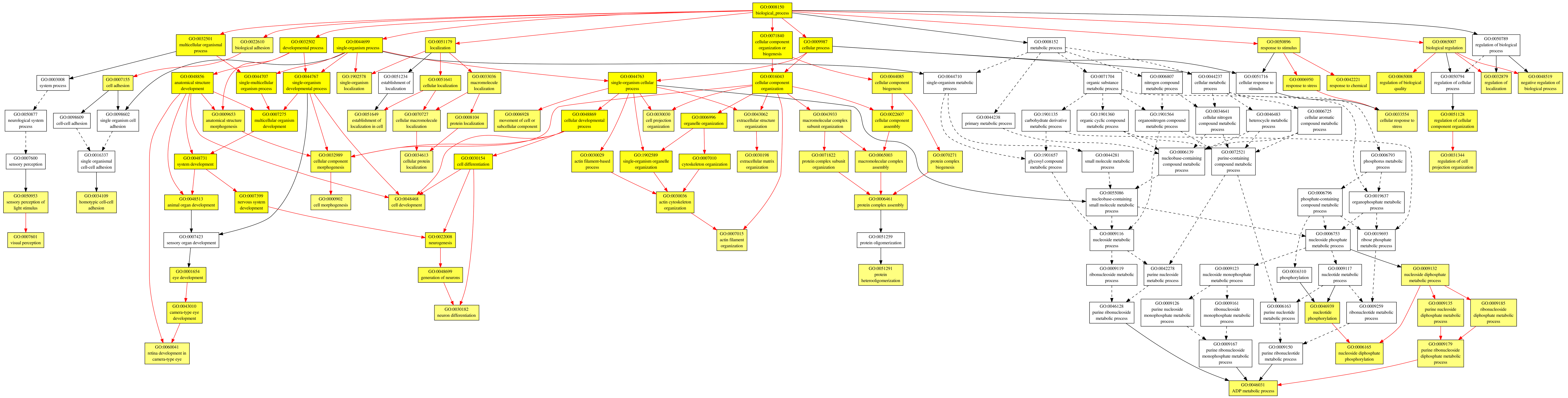

GO:0008150 biological_process
GO:0032501 multicellular organismal process
GO:0022610 biological adhesion
GO:0032502 developmental process
GO:0044699 single-organism process
GO:0051179 localization
GO:0071840 cellular component organization or biogenesis
GO:0009987 cellular process
GO:0008152 metabolic process
GO:0050896 response to stimulus
GO:0065007 biological regulation
GO:0050789 regulation of biological process
GO:0003008 system process
GO:0007155 cell adhesion
GO:0048856 anatomical structure development
GO:0044707 single-multicellular organism process
GO:0044767 single-organism developmental process
GO:1902578 single-organism localization
GO:0051234 establishment of localization
GO:0051641 cellular localization
GO:0033036 macromolecule localization
GO:0044763 single-organism cellular process
GO:0016043 cellular component organization
GO:0044085 cellular component biogenesis
GO:0044710 single-organism metabolic process
GO:0071704 organic substance metabolic process
GO:0006807 nitrogen compound metabolic process
GO:0044237 cellular metabolic process
GO:0051716 cellular response to stimulus
GO:0006950 response to stress
GO:0042221 response to chemical
GO:0065008 regulation of biological quality
GO:0050794 regulation of cellular process
GO:0032879 regulation of localization
GO:0048519 negative regulation of biological process
GO:0050877 neurological system process
GO:0098609 cell-cell adhesion
GO:0098602 single organism cell adhesion
GO:0009653 anatomical structure morphogenesis
GO:0007275 multicellular organism development
GO:0051649 establishment of localization in cell
GO:0070727 cellular macromolecule localization
GO:0008104 protein localization
GO:0006928 movement of cell or subcellular component
GO:0048869 cellular developmental process
GO:0030030 cell projection organization
GO:0006996 organelle organization
GO:0043062 extracellular structure organization
GO:0043933 macromolecular complex subunit organization
GO:0022607 cellular component assembly
GO:0044238 primary metabolic process
GO:1901135 carbohydrate derivative metabolic process
GO:1901360 organic cyclic compound metabolic process
GO:1901564 organonitrogen compound metabolic process
GO:0034641 cellular nitrogen compound metabolic process
GO:0046483 heterocycle metabolic process
GO:0006725 cellular aromatic compound metabolic process
GO:0033554 cellular response to stress
GO:0051128 regulation of cellular component organization
GO:0007600 sensory perception
GO:0016337 single organismal cell-cell adhesion
GO:0048731 system development
GO:0032989 cellular component morphogenesis
GO:0034613 cellular protein localization
GO:0030154 cell differentiation
GO:0030029 actin filament-based process
GO:1902589 single-organism organelle organization
GO:0007010 cytoskeleton organization
GO:0030198 extracellular matrix organization
GO:0071822 protein complex subunit organization
GO:0065003 macromolecular complex assembly
GO:0070271 protein complex biogenesis
GO:1901657 glycosyl compound metabolic process
GO:0044281 small molecule metabolic process
GO:0006139 nucleobase-containing compound metabolic process
GO:0072521 purine-containing compound metabolic process
GO:0006793 phosphorus metabolic process
GO:0031344 regulation of cell projection organization
GO:0050953 sensory perception of light stimulus
GO:0034109 homotypic cell-cell adhesion
GO:0048513 animal organ development
GO:0007399 nervous system development
GO:0000902 cell morphogenesis
GO:0048468 cell development
GO:0030036 actin cytoskeleton organization
GO:0006461 protein complex assembly
GO:0055086 nucleobase-containing small molecule metabolic process
GO:0006796 phosphate-containing compound metabolic process
GO:0019637 organophosphate metabolic process
GO:0007601 visual perception
GO:0007423 sensory organ development
GO:0022008 neurogenesis
GO:0007015 actin filament organization
GO:0051259 protein oligomerization
GO:0009116 nucleoside metabolic process
GO:0006753 nucleoside phosphate metabolic process
GO:0019693 ribose phosphate metabolic process
GO:0001654 eye development
GO:0048699 generation of neurons
GO:0051291 protein heterooligomerization
GO:0009119 ribonucleoside metabolic process
GO:0042278 purine nucleoside metabolic process
GO:0009123 nucleoside monophosphate metabolic process
GO:0016310 phosphorylation
GO:0009117 nucleotide metabolic process
GO:0009132 nucleoside diphosphate metabolic process
GO:0043010 camera-type eye development
GO:0030182 neuron differentiation
GO:0046128 purine ribonucleoside metabolic process
GO:0009126 purine nucleoside monophosphate metabolic process
GO:0009161 ribonucleoside monophosphate metabolic process
GO:0006163 purine nucleotide metabolic process
GO:0046939 nucleotide phosphorylation
GO:0009259 ribonucleotide metabolic process
GO:0009135 purine nucleoside diphosphate metabolic process
GO:0009185 ribonucleoside diphosphate metabolic process
GO:0060041 retina development in camera-type eye
GO:0009167 purine ribonucleoside monophosphate metabolic process
GO:0009150 purine ribonucleotide metabolic process
GO:0006165 nucleoside diphosphate phosphorylation
GO:0009179 purine ribonucleoside diphosphate metabolic process
GO:0046031 ADP metabolic process

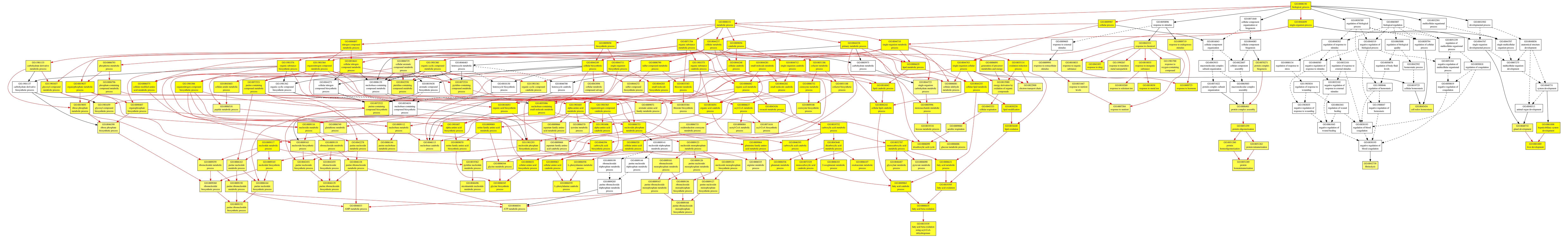

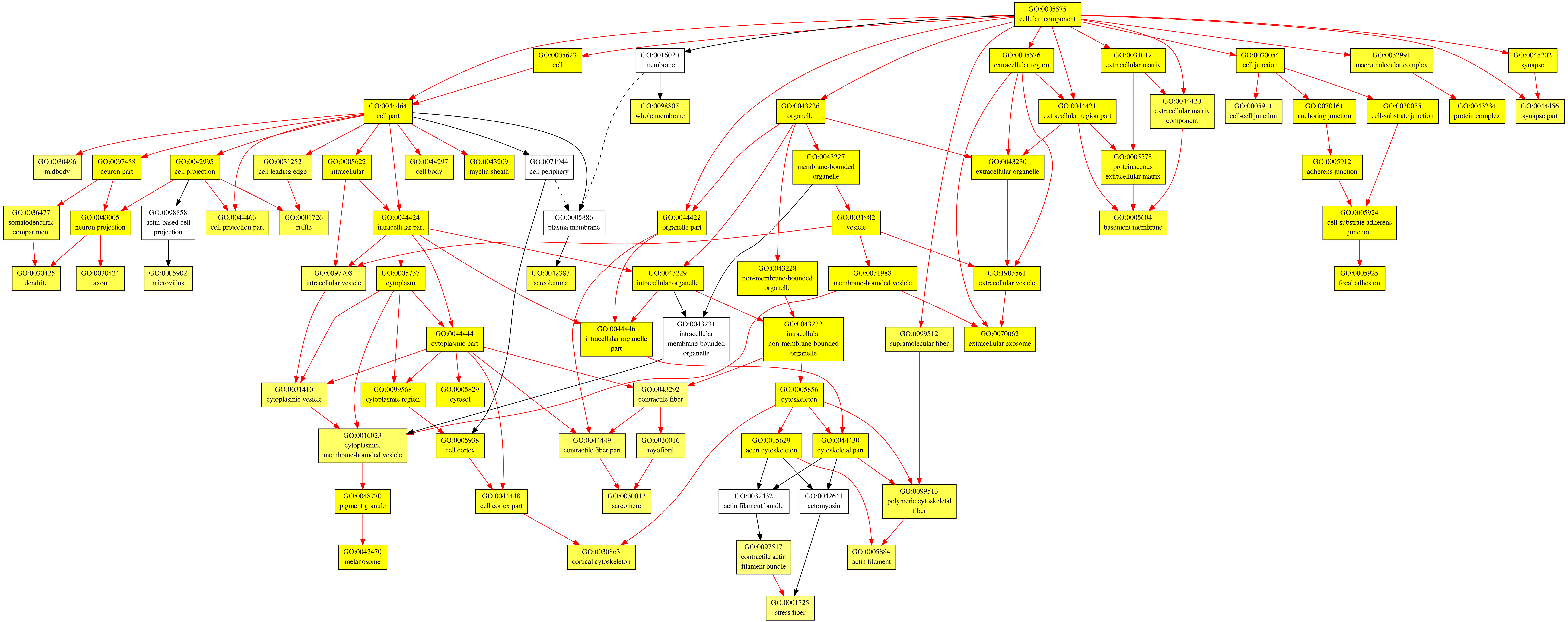
GO:0005575 cellular_component
GO:0005623 cell
GO:0016020 membrane
GO:0005576 extracellular region
GO:0031012 extracellular matrix
GO:0030054 cell junction
GO:0032991 macromolecular complex
GO:0045202 synapse
GO:0044464 cell part
GO:0098805 whole membrane
GO:0043226 organelle
GO:0044421 extracellular region part
GO:0044420 extracellular matrix component
GO:0005911 cell-cell junction
GO:0070161 anchoring junction
GO:0030055 cell-substrate junction
GO:0043234 protein complex
GO:0044456 synapse part
GO:0030496 midbody
GO:0097458 neuron part
GO:0042995 cell projection
GO:0031252 cell leading edge
GO:0005622 intracellular
GO:0044297 cell body
GO:0043209 myelin sheath
GO:0071944 cell periphery
GO:0043227 membrane-bounded organelle
GO:0043230 extracellular organelle
GO:0005578 proteinaceous extracellular matrix
GO:0005912 adherens junction
GO:0036477 somatodendritic compartment
GO:0043005 neuron projection
GO:0098858 actin-based cell projection
GO:0044463 cell projection part
GO:0001726 ruffle
GO:0044424 intracellular part
GO:0005886 plasma membrane
GO:0044422 organelle part
GO:0031982 vesicle
GO:0005604 basement membrane
GO:0005924 cell-substrate adherens junction
GO:0030425 dendrite
GO:0030424 axon
GO:0005902 microvillus
GO:0097708 intracellular vesicle
GO:0005737 cytoplasm
GO:0042383 sarcolemma
GO:0043229 intracellular organelle
GO:0043228 non-membrane-bounded organelle
GO:0031988 membrane-bounded vesicle
GO:1903561 extracellular vesicle
GO:0005925 focal adhesion
GO:0044444 cytoplasmic part
GO:0044446 intracellular organelle part
GO:0043231 intracellular membrane-bounded organelle
GO:0043232 intracellular non-membrane-bounded organelle
GO:0099512 supramolecular fiber
GO:0070062 extracellular exosome
GO:0031410 cytoplasmic vesicle
GO:0099568 cytoplasmic region
GO:0005829 cytosol
GO:0043292 contractile fiber
GO:0005856 cytoskeleton
GO:0016023 cytoplasmic, membrane-bounded vesicle
GO:0005938 cell cortex
GO:0044449 contractile fiber part
GO:0030016 myofibril
GO:0015629 actin cytoskeleton
GO:0044430 cytoskeletal part
GO:0048770 pigment granule
GO:0044448 cell cortex part
GO:0030017 sarcomere
GO:0032432 actin filament bundle
GO:0042641 actomyosin
GO:0099513 polymeric cytoskeletal fiber
GO:0042470 melanosome
GO:0030863 cortical cytoskeleton
GO:0097517 contractile actin filament bundle
GO:0005884 actin filament
GO:0001725 stress fiber

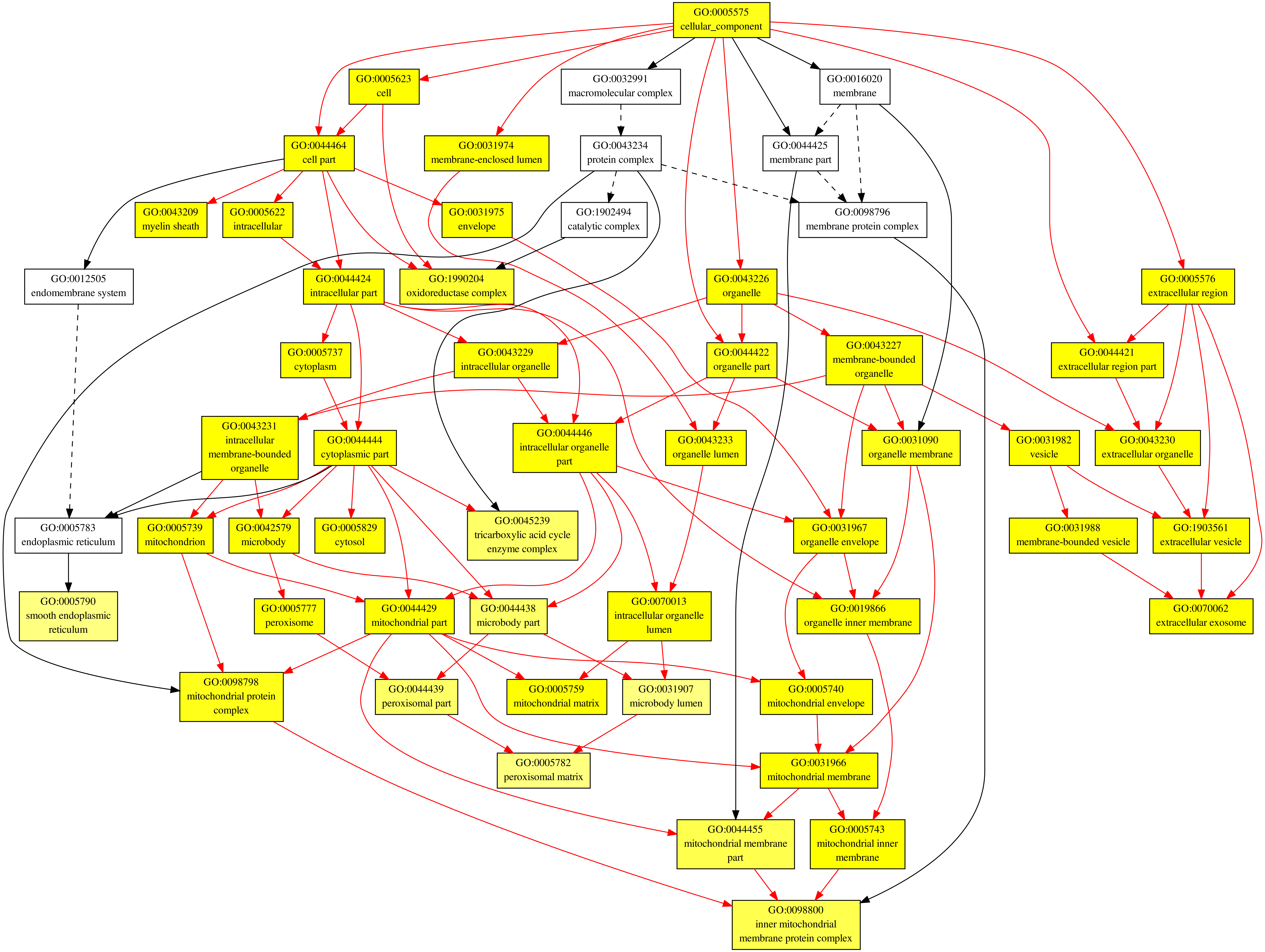
GO:0005575 cellular_component
GO:0005623 cell
GO:0032991 macromolecular complex
GO:0016020 membrane
GO:0044464 cell part
GO:0031974 membrane-enclosed lumen
GO:0043234 protein complex
GO:0044425 membrane part
GO:0043209 myelin sheath
GO:0005622 intracellular
GO:0031975 envelope
GO:1902494 catalytic complex
GO:0098796 membrane protein complex
GO:0012505 endomembrane system
GO:0044424 intracellular part
GO:1990204 oxidoreductase complex
GO:0043226 organelle
GO:0005576 extracellular region
GO:0005737 cytoplasm
GO:0043229 intracellular organelle
GO:0044422 organelle part
GO:0043227 membrane-bounded organelle
GO:0044421 extracellular region part
GO:0043231 intracellular membrane-bounded organelle
GO:0044444 cytoplasmic part
GO:0044446 intracellular organelle part
GO:0043233 organelle lumen
GO:0031090 organelle membrane
GO:0031982 vesicle
GO:0043230 extracellular organelle
GO:0005783 endoplasmic reticulum
GO:0005739 mitochondrion
GO:0042579 microbody
GO:0005829 cytosol
GO:0045239 tricarboxylic acid cycle enzyme complex
GO:0031967 organelle envelope
GO:0031988 membrane-bounded vesicle
GO:1903561 extracellular vesicle
GO:0005790 smooth endoplasmic reticulum
GO:0005777 peroxisome
GO:0044429 mitochondrial part
GO:0044438 microbody part
GO:0070013 intracellular organelle lumen
GO:0019866 organelle inner membrane
GO:0070062 extracellular exosome
GO:0098798 mitochondrial protein complex
GO:0044439 peroxisomal part
GO:0005759 mitochondrial matrix
GO:0031907 microbody lumen
GO:0005740 mitochondrial envelope
GO:0005782 peroxisomal matrix
GO:0031966 mitochondrial membrane
GO:0044455 mitochondrial membrane part
GO:0005743 mitochondrial inner membrane
GO:0098800 inner mitochondrial membrane protein complex

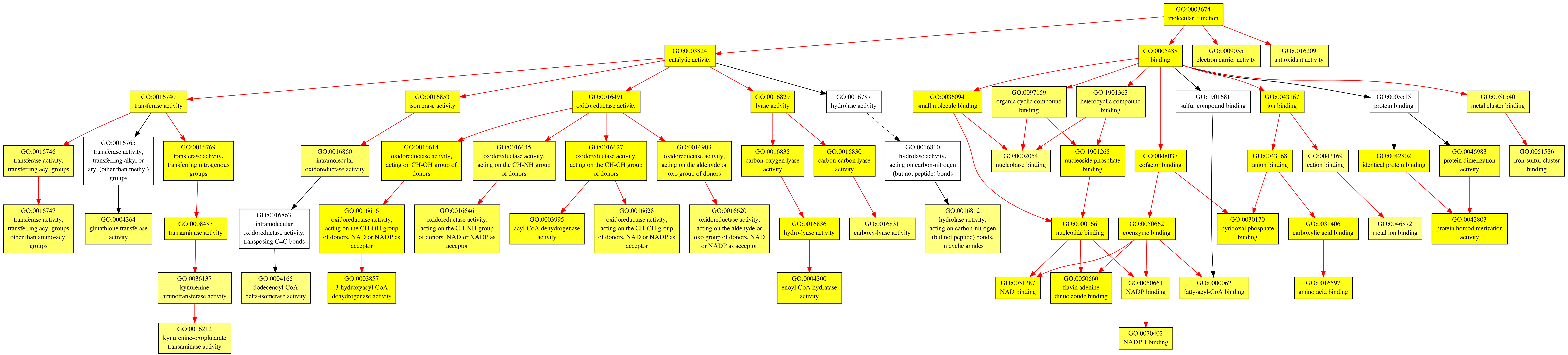
GO:0003674 molecular_function
GO:0003824 catalytic activity
GO:0005488 binding
GO:0009055 electron carrier activity
GO:0016209 antioxidant activity
GO:0016740 transferase activity
GO:0016853 isomerase activity
GO:0016491 oxidoreductase activity
GO:0016829 lyase activity
GO:0016787 hydrolase activity
GO:0036094 small molecule binding
GO:0097159 organic cyclic compound binding
GO:1901363 heterocyclic compound binding
GO:1901681 sulfur compound binding
GO:0043167 ion binding
GO:0005515 protein binding
GO:0051540 metal cluster binding
GO:0016746 transferase activity, transferring acyl groups
GO:0016765 transferase activity, transferring alkyl or aryl (other than methyl) groups
GO:0016769 transferase activity, transferring nitrogenous groups
GO:0016860 intramolecular oxidoreductase activity
GO:0016614 oxidoreductase activity, acting on CH-OH group of donors
GO:0016645 oxidoreductase activity, acting on the CH-NH group of donors
GO:0016627 oxidoreductase activity, acting on the CH-CH group of donors
GO:0016903 oxidoreductase activity, acting on the aldehyde or oxo group of donors
GO:0016835 carbon-oxygen lyase activity
GO:0016830 carbon-carbon lyase activity
GO:0016810 hydrolase activity, acting on carbon-nitrogen (but not peptide) bonds
GO:0002054 nucleobase binding
GO:1901265 nucleoside phosphate binding
GO:0048037 cofactor binding
GO:0043168 anion binding
GO:0043169 cation binding
GO:0042802 identical protein binding
GO:0046983 protein dimerization activity
GO:0051536 iron-sulfur cluster binding
GO:0016747 transferase activity, transferring acyl groups other than amino-acyl groups
GO:0004364 glutathione transferase activity
GO:0008483 transaminase activity
GO:0016863 intramolecular oxidoreductase activity, transposing C=C bonds
GO:0016616 oxidoreductase activity, acting on the CH-OH group of donors, NAD or NADP as acceptor
GO:0016646 oxidoreductase activity, acting on the CH-NH group of donors, NAD or NADP as acceptor
GO:0003995 acyl-CoA dehydrogenase activity
GO:0016628 oxidoreductase activity, acting on the CH-CH group of donors, NAD or NADP as acceptor
GO:0016620 oxidoreductase activity, acting on the aldehyde or oxo group of donors, NAD or NADP as acceptor
GO:0016836 hydro-lyase activity
GO:0016831 carboxy-lyase activity
GO:0016812 hydrolase activity, acting on carbon-nitrogen (but not peptide) bonds, in cyclic amides
GO:0000166 nucleotide binding
GO:0050662 coenzyme binding
GO:0030170 pyridoxal phosphate binding
GO:0031406 carboxylic acid binding
GO:0046872 metal ion binding
GO:0042803 protein homodimerization activity
GO:0036137 kynurenine aminotransferase activity
GO:0004165 dodecenoyl-CoA delta-isomerase activity
GO:0003857 3-hydroxyacyl-CoA dehydrogenase activity
GO:0004300 enoyl-CoA hydratase activity
GO:0051287 NAD binding
GO:0050660 flavin adenine dinucleotide binding
GO:0050661 NADP binding
GO:0000062 fatty-acyl-CoA binding
GO:0016597 amino acid binding
GO:0016212 kynurenine-oxoglutarate transaminase activity
GO:0070402 NADPH binding
